# Safety and efficacy of allogeneic umbilical cord red blood cell transfusion for children with severe anaemia in a Kenyan hospital: an open-label single-arm trial

**DOI:** 10.1016/S2352-3026(15)00005-8

**Published:** 2015-02-13

**Authors:** Oliver W Hassall, Johnstone Thitiri, Greg Fegan, Fauzat Hamid, Salim Mwarumba, Douglas Denje, Kongo Wambua, Kishor Mandaliya, Kathryn Maitland, Imelda Bates

**Affiliations:** a Centre for Geographic Medicine Research (Coast), Kenya Medical Research Institute (KEMRI)/Wellcome Trust Research Programme, Kilifi, Kenya; b Liverpool School of Tropical Medicine, Liverpool, UK; c Centre for Tropical Medicine and Global Health, University of Oxford, Oxford, UK; d Coast Provincial General Hospital, Mombasa, Kenya; e Regional Blood Transfusion Centre, Mombasa, Kenya; f Department of Paediatrics, Imperial College London, London, UK

## Abstract

**Background:**

In sub-Saharan Africa, children are frequently admitted with severe anaemia needing an urgent blood transfusion, but blood is often unavailable. When conventional blood supplies are inadequate, allogeneic umbilical cord blood could be a feasible alternative. The aim of this study was to assess the safety and efficacy of cord blood transfusion in children with severe anaemia.

**Methods:**

Between June 26, 2007, and May 20, 2008, 413 children needing an urgent blood transfusion were admitted to Kilifi District Hospital in Kenya. Of these, 87 children were eligible for our study—ie, younger than 12 years, no signs of critical illness, and haemoglobin 100 g/L or lower (if aged 3 months or younger) or 40 g/L or lower (if older than 3 months). Cord blood was donated at Coast Provincial General Hospital, Mombasa, and screened for transfusion-transmitted infections and bacterial contamination. Red blood cells were stored vertically at 2–6°C to enable sedimentation. After transfusion, children were monitored closely for adverse events and followed up for 28 days. The primary outcome measure was the frequency and nature of adverse reactions associated with the transfusion. Secondary outcomes were the changes in haemoglobin concentrations 24 h and 28 days after transfusion, compared with pretransfusion levels. This trial is registered on ISRCTN.com, number ISRCTN66687527.

**Findings:**

Of the 87 children eligible for the study, cord blood was unavailable for 24, six caregivers declined consent, and two children were withdrawn before transfusion. Therefore, 55 children received umbilical cord red blood cells from 74 donations. Ten (18%) children had ten serious adverse events and 43 (78%) had 94 adverse events; the most frequent adverse events were anaemia (n=14), weight loss (n=12), and vomiting (n=10). An independent expert panel judged none of these adverse events to be probably or certainly caused by the cord blood transfusion (one-sided 97·5% CI 0–6·5). Haemoglobin increased by a median of 26 g/L (IQR 21–31) 24 h after transfusion and by 50 g/L (10–68) a median of 29 days (28–35) after transfusion.

**Interpretation:**

These preliminary data suggest that cord blood could be an important supplementary source of blood for transfusion in children in sub-Saharan Africa. Further studies are needed to compare the safety and efficacy of cord blood with conventional adult-donated blood for transfusions. Challenges associated with cost, infrastructure, and scale up also need investigating.

**Funding:**

Wellcome Trust.

## Introduction

In Sub-Saharan Africa, babies in the first month of life have the highest risk of death, and the region has made little progress in reducing this high mortality rate.^1^ Severe anaemia is a major public health problem in sub-Saharan Africa, and children younger than 2 years are the most frequently affected. The prevalence of severe anaemia in children in hospital is 8–29%, with case fatality ranging from 8% to 18%.^2^ In children with severe uncompensated anaemia, blood transfusion can reduce mortality substantially.^3^ More than 50% of deaths happen within 4 h of admission, and early intervention and a source of safe blood are key components of the treatment of severe anaemia in childhood.^4^, ^5^ Supply of conventional blood for transfusion in sub-Saharan Africa is insufficient, with only an estimated 52% of demand being met and a shortfall of at least 2 million units a year.^6^, ^7^, ^8^

In situations when blood supply is limited, or young children need a substantial number of transfusions, the umbilical cord is a novel and potentially important source of blood.^9^, ^10^, ^11^ Use of cord blood might not only enable an increase in the number of small-volume transfusions but also reduce pressure on stocks of conventional adult-donated blood, thereby augmenting supplies for emergency transfusions for other vulnerable groups. In sub-Saharan Africa, a scarcity of blood for transfusion is implicated in 25% of maternal deaths due to haemorrhage.^12^

To test the feasibility of cord blood transfusion, we have established a cord blood donation programme on the labour ward at Coast Provincial General Hospital in Mombasa, Kenya. Previously, we have shown the acceptability to mothers of cord blood donation and transfusion, the feasibility of a two-stage informed consent process for cord blood donation, and the quality of variable volumes of whole cord blood stored in a fixed volume of anticoagulant preservative solution.^13^, ^14^ We also reported that, for cord blood obtained by our study team, the frequency of both bacterial contamination and seroreactivity for HIV, hepatitis B and C viruses, and syphilis compare favourably with those for conventional adult blood donated to the regional blood transfusion centre in Mombasa.^15^ To our knowledge, we report here the first clinical trial of allogeneic cord blood transfusion in children with severe anaemia. The aim of our study was to assess the safety and efficacy of umbilical cord red blood cell transfusion in children with severe anaemia.

## Methods

### Participants

We designed an open-label single-arm study with the aim to produce preliminary data for safety, harm, and haematological efficacy of umbilical cord red blood cell transfusion in children with severe anaemia. We recruited children younger than 12 years who were admitted for paediatric care at Kilifi District Hospital, Kenya. We designed our eligibility criteria to identify children for whom a transfusion would provide clinical benefit, based on WHO clinical guidelines, but exclude those who were critically ill.^16^

Children were eligible for inclusion in the study if they had severe anaemia (haemoglobin ≤100 g/L in babies aged 3 months or younger, or ≤40 g/L in children older than 3 months) and the attending clinician requested a blood transfusion. We excluded children with any of these clinical features of critical illness: coma (Blantyre coma scale ≤2); prostration; shock; deep (acidotic) breathing; and hyperbilirubinaemia requiring exchange transfusion. Furthermore, we judged children ineligible for the study if they had received a previous cord blood transfusion as part of this trial or were already enrolled in another intervention trial. We only enrolled a child into the study if sufficient cord blood was available.

All caregivers of participating children gave written informed consent. The Kenyan national ethics committee and the research ethics committee of the Liverpool School of Tropical Medicine (UK) reviewed and approved the study protocol.

### Procedures

We obtained cord blood from placentas donated at Coast Provincial General Hospital in Mombasa. We screened all donations for HIV, hepatitis B and C viruses, and syphilis, as described previously.^15^ All samples were quarantined until they were screened for bacterial contamination, which we did by incubating a 4 mL sample of cord blood in 40 mL of brain-heart infusion at 37°C for 48 h, as described previously.^15^, ^17^ Screening was by microscopic examination of a Gram-stained smear. We transported units of screened cord blood by road to Kilifi (a distance of 50 km) at 2–6°C. We stored samples vertically in racks at 2–6°C to enable sedimentation of red blood cells.

We used an electronic database to record the volume, haemoglobin concentration, and blood group of cord blood units. As soon as a blood transfusion was requested for an eligible child, we referred to this database to ascertain whether sufficient cord blood was available. The hospital clinical laboratory at Kilifi District Hospital used standard methods for blood grouping and cross-matching. At least 2·2 g/kg of haemoglobin was required for transfusion from a maximum of two group-identical or group-compatible cord blood units. Thus, we selected cord blood units based on estimated haemoglobin content, rather than volume. Furthermore, no child received a transfusion with more than 3·5 mL/kg of the preservative citrate phosphate dextrose adenine (CPDA-1).

Research staff from the Kenya Medical Research Institute (KEMRI)/Wellcome Trust research programme provided 24 h clinical cover at Kilifi District Hospital on the paediatric wards and paediatric high-dependency unit. At admission, all children underwent structured clinical assessment, including anthropometric measurements and standard laboratory investigations, such as estimation of haemoglobin concentration (Beckman Coulter, Villepinte, France), a blood film examination for malaria, and blood culture. We did haemoglobin electrophoresis retrospectively to detect haemoglobin S in children older than 3 months. Full investigation of the cause of severe anaemia was not part of the study protocol.

Before the cord blood transfusion, and for a period of 24 h afterwards, we admitted children to the paediatric high-dependency unit. In children with severe acute malnutrition (defined as a weight-for-height *Z* score less than −3 in children older than 3 months), we transfused umbilical cord red blood cells over a period of 3 h with a maximum permitted volume of 10 mL/kg, and we administered 1 mg/kg of furosemide intravenously at the start of the transfusion, according to clinical guidelines.^16^ For children without severe acute malnutrition, we transfused umbilical cord red blood cells over a period of 4 h with a maximum permitted volume of 20 mL/kg, and we did not administer furosemide.

For the first 2 h of the cord blood transfusion, we did continuous physiological monitoring. We recorded temperature, pulse rate, respiration rate, oxygen saturation, and blood pressure before transfusion, 15 min after the start, and every 30 min thereafter. After 2 h, we obtained a blood sample to estimate serum potassium (iLyte ion selective electrode analyser; Instrumentation Laboratory, USA) and calcium (Selectra E; Vital Scientific, Netherlands). We took a blood sample for haemoglobin estimation 24 h after the start of the cord blood transfusion, unless a haemoglobin measurement was requested for clinical management of the child before this time, in which case we used this result. We obtained a further blood sample for haemoglobin estimation from children who returned for follow-up after 28 days.

When a child was discharged from hospital, we gave their caregiver the cost of the fare home and the return fare back to the hospital, and we invited the caregiver to bring the child to the hospital 28 days after the cord blood transfusion. We encouraged them to come back to the hospital before then if they had any concerns about their child. We recorded details of the location of the child's home. Children who returned to hospital at 28 days had a structured clinical assessment. For those who did not attend hospital, a fieldworker followed up at home, confirming whether the child was alive and well by either direct observation or discussion with an adult family member; caregivers were also encouraged to bring their child to the hospital for a full review.

Detection of adverse reactions was a two-stage process comprising rigorous surveillance of adverse events (monitoring of harm) and an independent expert judgment about the relation of the adverse event to the umbilical cord red blood cell transfusion (assessment of imputability). To capture adverse events, a clinician reviewed every child and did a study-specific structured clinical assessment 2 h after the end of the transfusion, 24 h after the end of the transfusion, and at discharge from hospital. For the remainder of the child's time in hospital, monitoring of harm was by review of the daily clinical record kept by the attending clinicians. We defined serious adverse reactions as any serious adverse event (ie, any untoward medical occurrence that is fatal, life-threatening, disabling, prolongs admission, or results in admission)^18^ that was judged probably or certainly related to the transfusion. We defined adverse reactions as any adverse event (ie, any untoward medical occurrence)^18^ judged probably or certainly related to the transfusion.

The principal investigator (OWH) and an independent local safety monitor (a skilled consultant paediatrician) reviewed all serious adverse events and prepared a case summary, which was sent to the safety review committee. This committee consisted of three paediatricians who were independent of the study, with extensive experience of the clinical care of children in sub-Saharan Africa. The safety review committee and the local safety monitor reached consensus about the probability that a serious adverse event was caused by the transfusion of umbilical cord red blood cells and assigned the event an imputability score based on an established four-point scale, ranging from unlikely (0) to certain (3).^19^ A study clinician (FH) and the principal investigator (OWH) reviewed all other (non-serious) adverse events, which were described according to an established adverse reaction nomenclature.^20^ They used the same four-point imputability scale^19^ to score the probability of a causative relation of an adverse event with umbilical cord red blood cell transfusion. A summary of these adverse events was reviewed by a safety review committee and the local safety monitor.

The trial was to be stopped in the event of a suspected unexpected serious adverse reaction and not recommenced until a full review had been undertaken by the safety review committee and their recommendations seen and approved by both research ethics committees. Moreover, in the event of a serious adverse event, the safety review committee advised whether they felt that the trial should continue with no change to the protocol, continue with a change to the protocol, or be stopped.

### Outcomes

The primary outcome measure was the frequency and nature of adverse reactions occurring during or within at least 28 days of the umbilical cord red blood cell transfusion. The secondary outcome measure was the median change from pretransfusion levels in haemoglobin concentrations 24 h and 28 days after cord blood transfusion.

In the event of an adverse reaction after a cord blood transfusion (which comprised a maximum of two blood units), imputability could not be assigned to one of the two units. Therefore, the denominator for the primary outcome was the number of children receiving a transfusion. Children who received a subsequent conventional blood transfusion during the follow-up period were included in the analysis of the primary outcome, because these transfusions could themselves be evidence of harm related to a cord blood transfusion. However, children receiving a conventional blood transfusion were not included in the analysis of haemoglobin change at 28 days, because subsequent transfusions would have confounded the effect of the cord blood transfusion.

### Statistical analysis

We estimated from previous data that 100 children fulfilling the eligibility criteria for the trial would be admitted to Kilifi District Hospital during a period of 1 year and that cord blood would be available and consent for a transfusion given for 40–80% of these children. Thus, during 1 year of study, 40–80 children might be recruited to the trial. We intended to run the trial for 1 year; therefore, we set these numbers as a minimum and maximum sample size. The appendix (p 1) shows estimates for the frequency of adverse reactions at these minimum and maximum sample sizes.

We expressed binary data as a percentage with 95% CIs where appropriate. When event frequencies were zero, we calculated a one-sided 97·5% CI with a lower limit of zero. We summarised continuous data with medians and range or IQR. We compared noted differences in continuous data with non-parametric statistics (Wilcoxon rank-sum test).

This trial is registered on ISRCTN.com, number ISRCTN66687527.

### Role of the funding source

The funder had no role in study design, data collection, data analysis, data interpretation, or writing of the report. The corresponding author had full access to all data in the study and had final responsibility for the decision to submit for publication.

## Results

Between June 26, 2007, and May 20, 2008, 413 children admitted to Kilifi District Hospital, Kenya, needed a blood transfusion; of these, 87 were eligible for our trial (figure). An umbilical cord red blood cell donation of either sufficient haemoglobin content or the correct blood group was unavailable for 24 children, and the caregiver declined consent for six children. Thus, 57 children were recruited to the study. Two participants were withdrawn before umbilical cord red blood cell transfusion. In one case, the laboratory made an error during compatibility testing and no further cord blood was available. In the second case, clinical review soon after recruitment showed deep breathing, which was an exclusion criterion.

55 children received umbilical cord red blood cells from 74 cord blood donations. Of these, 24 children were aged 3 months or younger and 31 were older than 3 months (table 1); the median age of children in the study was 12 months (range 2 days to 5 years 8 months). Children weighed between 1·1 kg and 14·5 kg (median, 5·3 kg). The median weight-for-height *Z* score was 1·9 (range −4·4 to −0·9), and seven children had severe acute malnutrition. All children with severe acute malnutrition received 10 mL/kg of umbilical cord red blood cells; for those without severe acute malnutrition, the median volume transfused was 14 mL/kg (range 10–20).

In children aged 3 months or younger, pretransfusion haemoglobin was a median of 87 g/L (range 55–100, IQR 78–92). In children older than 3 months, median pretransfusion haemoglobin was 32 g/L (range 19–40, IQR 27–38). In all children, within a median of 24 h (IQR 17–24) of the cord blood transfusion, haemoglobin had risen by a median of 26 g/L (IQR 21–31; table 2). 33 children did not receive a further transfusion; after median follow-up of 29 days (IQR 28–35), the median rise in haemoglobin was 50 g/L (IQR 10–68). In children aged 3 months or younger, haemoglobin rose by a median of 5 g/L (IQR 2–12) at a median of 29 days (28–36) of follow-up, compared with a median increase of 61 g/L (53–82) in children older than 3 months (median follow-up 30 days [28–35]; p<0·0001).

In the seven children with severe acute malnutrition (all older than 3 months), who received a maximum of 10 mL/kg umbilical cord red blood cells, the median rise in haemoglobin 24 h after transfusion was 21 g/L (IQR 20–29). In 24 children older than 3 months without severe acute malnutrition, who received between 10 mL/kg and 16 mL/kg umbilical cord red blood cells (median 13 mL/kg), the median rise in haemoglobin 24 h after transfusion was 26 g/L (IQR 22–31; p=0·15). Five of seven children with severe acute malnutrition had haemoglobin measured at 28-day follow-up; the median rise in haemoglobin in this subgroup was 81 g/L (IQR 78–82), compared with 59 g/L (IQR 53–68) in 15 of 24 children without severe acute malnutrition for whom haemoglobin concentration was measured at follow-up.

Of the 55 children who received an umbilical cord red blood cell transfusion, ten had a serious adverse event (one event per child) and 43 children had 94 adverse events (table 3; appendix p 2) The most frequent adverse events were anaemia (n=14), weight loss (n=12), and vomiting (n=10). In no case was transfusion of umbilical cord red blood cells judged probably or certainly implicated and, thus, the frequency of serious adverse reactions and adverse reactions was 0% (one-sided 97·5% CI 0–6·5).

Of the ten serious adverse events recorded, four were new signs of critical illness (deep breathing or prostration) noted during the pretransfusion assessment, before transfusion of umbilical cord red blood cells. Therefore, the cord blood transfusion was excluded as a potential cause of these serious adverse events. Of the remaining six serious adverse events, one child died, three events were judged life-threatening, and two resulted in admission after discharge (table 4).

## Discussion

Our findings show that, in a population of children from Kenya who were admitted to hospital with severe anaemia, transfusion of sedimented red blood cells from umbilical cord donations was safe and efficacious. No serious adverse events or adverse events were certainly or probably attributable to cord blood transfusion. Furthermore, the haemoglobin concentration after transfusion rose by 26 g/L after 24 h and by 50 g/L at about 28 days. Our findings accord with previous scant data for allogeneic cord-blood transfusion.^9^

Although we excluded children with signs of critical illness at the time of study recruitment, adverse events were recorded in many participants, which were unrelated to the cord blood transfusion. In four children, signs of critical illness were detected at the clinical assessment undertaken just before cord blood transfusion. To withdraw these critically ill children from the study at that stage, and to secure and cross-match adult-donated blood, would have introduced an unacceptable delay in their management. This difficulty highlights the challenge of undertaking studies focusing on safety and harm in children admitted to hospital in sub-Saharan Africa. Robust monitoring frameworks are needed to identify potential associations between the effects of an intervention and other confounding factors. A weakness of our study is that, for children who did not attend the hospital for follow-up at 28 days, no structured clinical assessment was done. However, all children were followed up in the community by a non-clinical fieldworker, and the death of one participant was identified in this way.

The rise in haemoglobin recorded 24 h after cord blood transfusion (median 26 g/L) accords with estimates based on the haemoglobin content of transfused blood and the circulating volume of children: for a child with a circulating volume of 80 mL/kg, transfusion of 2·2 g/kg of haemoglobin might be expected to raise the haemoglobin concentration by 28 g/L. However, although cord blood units were selected for transfusion based on an estimation of the unit haemoglobin content, we cannot ascertain from these data how much haemoglobin was actually issued and transfused.

The significant rise in haemoglobin 28 days after transfusion in children older than 3 months, compared with infants aged 3 months or younger, accords with previous data from Kilifi and other sites in east Africa.^3^, ^4^, ^21^, ^22^ However, increases in haemoglobin over a similar period have also been seen in children with severe anaemia who do not receive a transfusion,^3^, ^21^, ^22^ which highlights the importance of other strategies to manage severe anaemia—eg, treatment of infection, use of anthelmintics and haematinics, and diet. The relative importance of these interventions will depend on the cause of anaemia, which we did not investigate here.

Infants younger than 3 months in our study were likely to have very different reasons for their anaemia compared with the older children—eg, many infants were presumed to have anaemia of prematurity. Several of these children needed further blood transfusions and, in those who did not, the effect of one umbilical cord red blood cell transfusion at 28 days was much more modest (5 g/L). However, the number of young infants who were eligible for a cord blood transfusion is noteworthy. This group of patients has a high burden of mortality in sub-Saharan Africa and potentially might benefit substantially from more evidence about the role of transfusion in prevention of high death rates.^1^ These young children might benefit in particular from the availability of cord blood for transfusion, because they only need small volumes of blood.

The microbiological safety of cord blood provided by the donation programme that we have established at Coast Provincial General Hospital in Mombasa compares favourably with that of conventional blood from the same setting.^15^ Mothers who donate their infants' umbilical cord blood are selected rigorously (including self-reporting of antenatal testing for syphilis and HIV), and aseptic cord blood collection is done by trained fieldworkers and not the midwives who manage the deliveries.^14^, ^15^ Furthermore, all cord blood donations in this study were screened for bacterial contamination. These rigorous techniques can be difficult to replicate outside of a research setting without additional resources.

Our findings suggest that further trials of umbilical cord red blood cell transfusions are warranted (panel), but the challenges of doing such trials and the barriers to potential scale up of such an intervention should not be underestimated. Attributing effects to the intervention is difficult in such a sick group of children. Despite this limitation, further clinical trials should also include children with signs of critical illness who potentially have the most to gain from an improved blood supply. The infrastructure and training needed to set up collection and administration of umbilical cord blood is complex, and such trials would need meticulous monitoring during and after the transfusion. Poor haemovigilance systems in these settings means that very little is known about the harms associated with conventional blood transfusion, which would be the comparator group in such trials.^7^

Several improvements and additions could be made to the design of future trials. Better characterisation of the cause of anaemia could be included, in addition to assessment of any correlation with benefits and harms of cord blood transfusion. Immunological and genetic testing could be done to compare rates of alloimmunisation and microchimerism. Finally, operational analyses could be done to compare the availability of cord blood and adult-donated blood for urgent transfusion in children and to look at how using cord blood for transfusions in children affects the blood supply for adults who need larger volumes of transfused blood.

In settings where demand for low-volume transfusions for children is high and supplies of conventional blood are low, umbilical cord blood could be a safe and effective supplementary source of blood for transfusion. Further trials comparing cord blood with conventional adult-donated blood transfusions are merited.

## Figures and Tables

**Figure:**
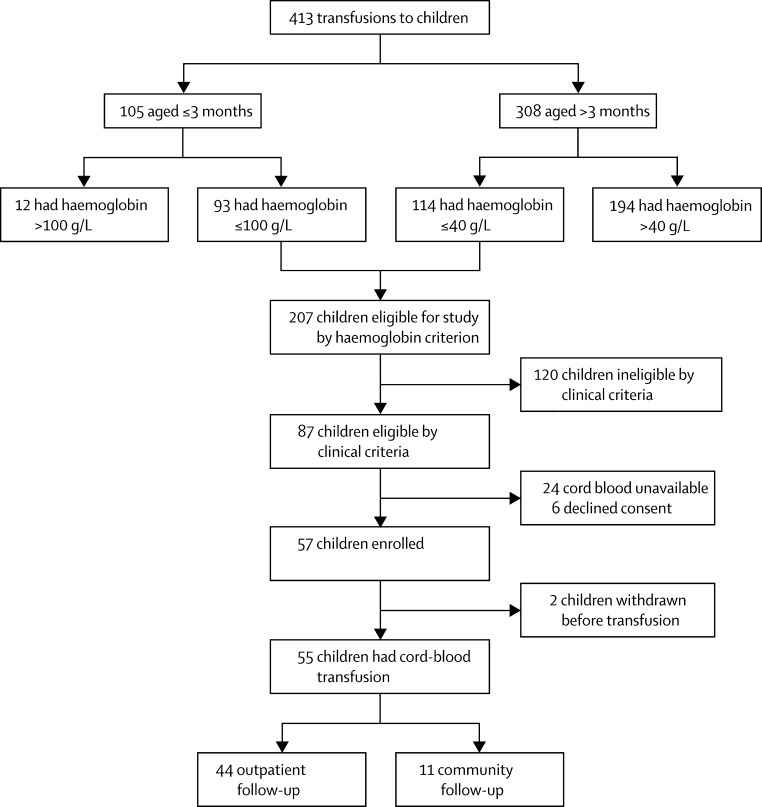
Study profile

**Table 1:** Selected characteristics of children at admission

	**Infants aged ≤3 months (n=24)**	**Infants aged >3 months (n=31)**	**Total (n=55)**
Girls/boys	12/12	15/16	27/28
Sickle-cell genotype SS	..	6	6
Malaria parasites present	0	6	6
Weight-for-height *Z* score less than −3	..	7	7
Preterm*	14	..	14
Weight (kg)	1·6 (1·1–4·9)	8·6 (4·7–14·5)	5·3 (1·1–14·5)
Pretransfusion haemoglobin concentration (g/L)	87 (55–100)	32 (19–40)	40 (19–100)

Data are either number of participants or median (range).

* Born before 37 weeks of pregnancy was completed.

**Table 2:** Haemoglobin concentration in children receiving umbilical cord red blood cell transfusions, stratified by age

	**Before transfusion**	**Within 24 h**	**From 24 h to 28 days**
**Age ≤3 months**			
Number of children	24	24	13
Haemoglobin concentration (g/L)	87 (78–92)	114 (105–124)	92 (84–99)
Change in haemoglobin concentration (g/L)	..	27 (22–38)	5 (2–12)
**Age >3 months**			
Number of children	31	31	20
Haemoglobin concentration (g/L)	32 (27–38)	58 (53–62)	93 (83–110)
Change in haemoglobin concentration (g/L)	..	26 (21–31)	61 (53–82)
**All children**			
Number of children	55	54*	33†
Haemoglobin concentration (g/L)	40 (31–82)	65 (57–113)	90 (84–102)
Change in haemoglobin concentration (g/L)	..	26 (21–31)	50 (10–68)

Data are median (IQR), unless otherwise stated.

* Excludes one child for whom consent for a blood test was declined.

† Excludes 22 children: ten received further transfusions; 11 were followed up in the community; and one was not bled in error.

**Table 3:** Serious adverse events and adverse events in children receiving umbilical cord red blood cell transfusions

		**Serious adverse events (n=10)**	**Adverse events (n=94)**
Children (n)		10	43
Event timing			
	Before transfusion	4	4
	During transfusion and within 24 h afterwards	1	12
	From 24 h to 28 days after transfusion	5	78
Indicator of severity			
	Fatal	1	N/A
	Life-threatening	7	N/A
	Admission	2	N/A
Imputability level*			
	Not assessable	0	0
	Excluded (0)	4	15
	Unlikely (0)	3	76
	Possible (1)	3	3
	Likely/probable (2)	0	0
	Certain (3)	0	0

N/A=not applicable.

* Numbers in parentheses refer to four-point imputability score.

**Table 4:** Serious adverse events within 1 month of umbilical cord red blood cell transfusion

	**Time after transfusion, and description of serious adverse event**	**Indicator of severity**	**Comments**	**Imputability level (score)**
Girl, aged 13 months	Acute leukaemia, probably acute lymphoblastic leukaemia	Fatal	Diagnosis of leukaemia made by microscopic examination of a peripheral blood film taken before cord blood transfusion but reported afterwards; child was referred to regional hospital but discharged against medical advice and taken home; child was pale, sick-looking, and febrile at discharge; child's grandfather reported she died the day after arriving home, about 1 week after cord blood transfusion	Unlikely (0)
Boy, aged 2 days	At 7 days, abdominal distension, respiratory distress, jaundice	Life-threatening	Infant was born preterm (estimated gestation, 30 weeks; birthweight, 1440 g) with probable neonatal sepsis; full recovery after intervention with broad-spectrum antibiotics, nil by mouth, intravenous fluid, and phototherapy	Unlikely (0)
Boy, aged 4 years	At 28 days, anaemia	Admission	Child had known sickle-cell disease; was discharged well 3 days after cord blood transfusion (haemoglobin 62 g/L) with haematinics; returned for follow-up at 21 days (haemoglobin 66 g/L); at 28-day follow-up, haemoglobin was 41 g/L; child admitted for conventional blood transfusion; discharged next day (haemoglobin 61 g/L)	Unlikely (0)
Boy, aged 24 days	After 26 h, diarrhoea, dehydration, and metabolic acidosis	Life-threatening	Preterm infant (estimated gestation, 28 weeks; birthweight, 1080 g) with probable neonatal sepsis; full recovery after intervention with oxygen, broad-spectrum antibiotics, and intravenous fluid	Possible (1)
Girl, aged 9 days	After 14 h, sepsis, pneumonia, and apnoea events	Life-threatening	Preterm infant (estimated gestation, 30 weeks; birthweight, 1200 g); pneumonia was confirmed by radiography; full recovery after intervention with broad-spectrum antibiotics, oxygen, aminophylline, and blood transfusion	Possible (1)
Girl, aged 5 days	At 28 days, anaemia	Admission	Baby had a low birthweight (probable prematurity), neonatal jaundice, and possible sepsis; was discharged well 3 days after cord blood transfusion (haemoglobin 115 g/L); at 28-day follow-up, haemoglobin was 62 g/L; admission offered but declined; child presented again 1 week later and was admitted (haemoglobin 84 g/L); conventional blood transfusion given once donor found (initially no blood available); discharged next day with haematinics	Possible (1)

Ages of children are at enrolment to the study.

## Panel Research in context

**Systematic review**

A review of published work relating to cord blood transfusion was done before the study (April, 2007) and repeated before submission (September, 2014). We searched PubMed with the following search query: (“cord blood”[Title/Abstract] OR “placental blood”[Title/Abstract] AND “transfusion”[Title/Abstract]); we did not restrict by date, language, or article type. The reference lists of articles identified were also screened for relevant titles. Allogeneic cord blood transfusion was first reported in the 1930s, before the advent of modern blood transfusion services.^23^ Subsequently, most research and clinical activity relating to cord blood transfusion has concerned autologous cord blood transfusion in preterm neonates.^11^ In India, a series of about 200 mainly elderly patients with chronic and terminal disease has received allogeneic cord blood transfusions.^9^, ^11^ No adverse reactions were reported. To our knowledge, our study is the first clinical trial of allogeneic cord blood transfusion in children.

**Interpretation**

In children with severe anaemia in sub-Saharan Africa, transfusion of sedimented red blood cells obtained from umbilical cord blood has a low probability of adverse events. Haemoglobin recovery after cord blood transfusion is within expected limits. Umbilical cord blood could be a safe and efficacious supplementary source of blood for transfusion when demand for low-volume transfusions for children is high and supplies of conventional blood are limited. Further work needs to be undertaken by clinical researchers to establish the safety and efficacy of cord blood transfusion compared with conventional blood transfusion. Additional research is also needed on the operational aspects of cord blood collection, including costs, the effect on conventional blood supply, and scalability.

## References

[1] You D, Bastian P, Wu J, Wardlaw T, on behalf of the United Nations Inter-agency Group for Child Mortality Estimation (2013). Levels and trends in child mortality. http://www.childinfo.org/files/Child_Mortality_Report_2013.pdf.

[2] WHO (2001). The prevention and management of severe anaemia in children in malaria-endemic regions of Africa: a review of research.

[3] Lackritz EM, Hightower AW, Zucker JR (1997). Longitudinal evaluation of severely anemic children in Kenya: the effect of transfusion on mortality and hematologic recovery. AIDS.

[4] English M, Ahmed M, Ngando C, Berkley J, Ross A (2002). Blood transfusion for severe anaemia in children in a Kenyan hospital. Lancet.

[5] Lackritz EM, Campbell CC, Ruebush TK, Hightower AW, Wakube W, Were JBO (1992). Effect of blood transfusion on survival among children in a Kenyan hospital. Lancet.

[6] Allain JP, Owusu-Ofori S, Bates I (2004). Blood transfusion in sub-Saharan Africa. Transfus Altern Transfus Med.

[7] Tagny CT, Mbanya D, Tapko JB, Lefrere JJ (2008). Blood safety in Sub-Saharan Africa: a multi-factorial problem. Transfusion.

[8] Tapko JB, Sam O, Diarra-Nama AJ (2007). Status of blood safety in the WHO African region: report of the 2004 survey.

[9] Bhattacharya N (2005). Placental umbilical cord whole blood transfusion: a safe and genuine blood substitute for patients of the under-resourced world at emergency. J Am Coll Surg.

[10] Hassall O, Bedu-Addo G, Adarkwa M, Danso K, Bates I (2003). Umbilical-cord blood for transfusion in children with severe anaemia in under-resourced countries. Lancet.

[11] Khodabux CM, Brand A (2009). The use of cord blood for transfusion purposes: current status. Vox Sang.

[12] Bates I, Chapotera GK, McKew S, van den Broek N (2008). Maternal mortality in sub-Saharan Africa: the contribution of ineffective blood transfusion services. BJOG.

[13] Hassall O, Ngina L, Kongo W (2008). The acceptability to women in Mombasa, Kenya, of the donation and transfusion of umbilical cord blood for severe anaemia in young children. Vox Sang.

[14] Hassall O, Maitland K, Fegan G (2010). The quality of stored umbilical cord and adult-donated whole blood in Mombasa, Kenya. Transfusion.

[15] Hassall OW, Thitiri J, Fegan G (2012). The microbiologic safety of umbilical cord blood transfusion for children with severe anemia in Mombasa, Kenya. Transfusion.

[16] WHO (2005). Pocket book of hospital care for children: guidelines for the management of common illnesses with limited resources.

[17] Hassall O, Maitland K, Pole L (2009). Bacterial contamination of pediatric whole blood transfusions in a Kenyan hospital. Transfusion.

[18] EMEA (2002). ICH Guideline for Good Clinical Practice.

[19] MHRA UK blood safety and quality regulations 2005: implementation of the EU blood safety directive—background and guidance on reporting serious adverse events and serious adverse reactions. http://www.mhra.gov.uk/home/groups/dts-aic/documents/websiteresources/con2022523.pdf.

[20] FDA (1995). COSTART: coding symbols for thesaurus of adverse reaction terms.

[21] Akech SO, Hassall O, Pamba A (2008). Survival and haematological recovery of children with severe malaria transfused in accordance to WHO guidelines in Kilifi, Kenya. Malaria J.

[22] Holzer BR, Egger M, Teuscher T, Koch S, Mboya DM, Davey Smith G (1993). Childhood anemia in Africa: to transfuse or not transfuse?. Acta Trop.

[23] Howkins J, Brewer HF (1939). Placental blood for transfusion. Lancet.

