# Supplementary material for: Safety and efficacy of allogeneic umbilical cord red blood cell transfusion for children with severe anaemia in a Kenyan hospital: an open-label single-arm trial
Source: Lancet Haematol. 2015 Feb 13;2(3):e101–7. doi: 10.1016/S2352-3026(15)00005-8 (PMC4722332; doi:10.1016/S2352-3026(15)00005-8)
Supplement: Supplementary appendix [file mmc1.pdf]

THE LANCET Haematology

Supplementary appendix

This appendix formed part of the original submission and has been peer reviewed. We post it as supplied by the authors.

Supplement to: Hassall OW, Thitiri J, Fegan G, et al. Safety and efficacy of allogeneic umbilical cord red blood cell transfusion for children with severe anaemia in a Kenyan hospital: an open-label single-arm trial. *Lancet Haematol* 2015; published online Feb 13. http://dx.doi.org/10.1016/ S2352-3026(15)00005-8.

Webappendix

Table 1 The frequency and confidence intervals of serious adverse reactions at the minimum and maximum predicted sample sizes

		Children transfused	
		40	80
Serious adverse reactions	*0	0.0<br>0.0-8.8	0.0<br>0.0-4.5
	1	2.5<br>0.1-13.2	1.3<br>0.0-6.8
	2	5.0<br>0.6-16.9	2.5<br>0.3-8.7
	3	7.5<br>1.6-20.4	3.8<br>0.8-10.6
	4	10.0<br>2.8-23.7	5.0<br>1.4-12.3
	5	12.5<br>4.2-26.8	6.3<br>2.1-14.0

* One-sided, 97.5% confidence intervals (with a lower limit of zero) are given for event frequencies of zero. All others are 95% confidence intervals.

Table 2 Description and frequency of selected (non-serious) adverse events occurring within one month of umbilical cord red cell transfusion*

	Imputability level (score)			
	Excluded (0)	Unlikely (0)	Possible (1)	All
Anaemia	1	11	2	14
Weight loss†	1	11	0	12
Vomiting§	3	7	0	10
Rash	0	8	0	8
Fever	0	5	0	5
Flu-like symptoms	0	3	0	3
Splenomegaly	0	3	0	3
Conjunctivitis	0	2	0	2
Otitis externa	0	2	0	2
Pneumonia	0	2	0	2
Stools hard	0	2	0	2
Hypocalcaemia	0	0	1	1

* In total there were 94 adverse events. Shown here are those with a frequency of two or more (64 adverse events) and/or those with an imputability level of 1 (3 adverse events) (see text for explanation)
† Includes one instance of no weight gain
§ Includes one instance of vomiting and diarrhoea
